# Inflammatory indexes are not associated with sarcopenia in Chinese community-dwelling older people: a cross-sectional study

**DOI:** 10.1186/s12877-020-01857-5

**Published:** 2020-11-07

**Authors:** Tianjiao Tang, Lingling Xie, Lingling Tan, Xiaoyi Hu, Ming Yang

**Affiliations:** 1 Center of Gerontology and Geriatrics, West China Hospital, Sichuan University, No.37 Guoxue Lane, Chengdu, Sichuan China; 2 Precision Medicine Research Center, West China Hospital, Sichuan University, No. 37 Guoxue Lane, Chengdu, Sichuan China; 3 National Clinical Research Center for Geriatric Diseases at West China Hospital, Sichuan University, No. 37 Guoxue Lane, Chengdu, Sichuan China

**Keywords:** Inflammatory indexes, Sarcopenia, Older adults, Muscle wasting, Muscle atrophy

## Abstract

**Background:**

Inflammatory indexes (platelet-to-lymphocyte ratio [PLR], neutrophil-to-lymphocyte ratio [NLR], and lymphocyte-to-monocyte ratio [LMR]) are recently supposed to be the biomarkers of sarcopenia. We aimed to validate the association between these inflammatory indexes and sarcopenia in Chinese community-dwelling older people.

**Methods:**

We consecutively recruited community-dwelling older adults aged 60 years or older. The neutrophil, lymphocyte, monocyte, and platelet counts, and C-reactive protein (CRP) were tested using standard methods. Sarcopenia was defined according to different criteria: the Asian Working Group for Sarcopenia (AWGS), the updated version of AWGS (AWGS 2019), the European Working Group on Sarcopenia in Older People (EWGSOP), the updated version of EWGSOP (EWGSOP2), the International Working Group on Sarcopenia (IWGS), and the Foundation for the National Institutes of Health Sarcopenia Project (FNIH). Multiple logistic regression analysis was performed.

**Results:**

We included 384 participants. A total of 61 participants (15.9%) were diagnosed with sarcopenia according to the AWGS criteria. There was no significant difference in PLR, NLR, LMR, and CRP between the sarcopenia group and the non-sarcopenia group regardless of the diagnostic criteria. No significant association between PLR, NLR, LMR, and AWGS-defined sarcopenia was found (PLR per 1- standard deviation [SD]: adjusted odds ratio [OR] 1.09, 95% confidence interval [CI] 0.82 to 1.45; NLR per 1-SD: adjusted OR 0.96, 95% CI 0.71 to 1.30; LMR per 1-SD: adjusted OR 1.01, 95% CI 0.74 to 1.38). Similar results were found when sarcopenia was defined by different criteria and when PLR, NLR, LMR were treated as categorical variables.

**Conclusions:**

Our study did not support the utility of the inflammatory indexes (NLR, PLR, and LMR) as the biomarkers of sarcopenia in Chinese community-dwelling older people. However, considering the inflammatory indexes can be simply calculated from a routine blood test, further studies in different populations remain warranted.

**Supplementary Information:**

The online version contains supplementary material available at 10.1186/s12877-020-01857-5.

## Background

Sarcopenia was traditionally considered as a geriatric syndrome featured by loss of muscle mass and low muscle strength and/or low physical performance [1]. Recently, it has been formally recognized as a muscle disease with an international classification of disease, tenth revision, clinical modification (ICD-10-CM) Diagnosis Code [2, 3]. Sarcopenia is common among older people and is related to numerous adverse health outcomes, such as falls, functional decline, hospitalization, poor quality of life, and death [4, 5]. It also increases health economic burden and induces challenges for public health.

Although the pathogenesis of sarcopenia remains unclear, recent evidence shows that chronic low-grade inflammation may play an important role in the development of sarcopenia. For example, inflammation may harm skeletal muscle mass through direct catabolic effects or by causing reduced dietary nutrition intake (induction of anorexia) [6, 7].

Many inflammatory markers, such as interleukin- 6 (IL-6), C-reactive protein (CRP), and tumor necrosis factor α (TNF-α), have been linked with sarcopenia [6, 8]. However, these biomarkers are not routinely tested in community settings when patients have no clinical evidence of inflammation, therefore, this may limit their utility in clinical practice. The inflammatory indexes, i.e., platelet-to-lymphocyte ratio (PLR), neutrophil-to-lymphocyte ratio (NLR), and lymphocyte-to-monocyte ratio (LMR), are calculated based on the neutrophil, lymphocyte, monocyte, and platelet counts that are routinely tested in most clinical settings worldwide. They are easily measurable and inexpensive, and have been regarded as important indicators of systemic inflammation [9, 10] and serve as predictive biomarkers in patients with coronary heart disease [11], various malignancies [12], and neurodegenerative disease [13]. Recently, a case-control study showed that NLR was an independent predictor for sarcopenia in Turkish older people admitted to the outpatient clinics [14]. Additionally, PLR was reported to be a novel biomarker of sarcopenia in American older people [15]. These findings are valuable but need to be validated in different populations. We, therefore, reanalyzed the data of a cross-sectional study to investigate the possible relationship between these inflammatory indexes and sarcopenia in Chinese community-dwelling older people.

## Methods

### Study design and participants

We reanalyzed the data of a cross-sectional study conducted in Chengdu, China, between October to November 2017. The details of the study design were previously reported [16]. Briefly, community-dwelling older people aged 60 years or older were consecutively recruited. The exclusion criteria included individuals with clinical evidence of acute or chronic inflammation, severe mental illness, implanted pacemaker, clinically visible edema, unable to walk, severe renal failure, severe heart failure, and inability to communicate with interviewers. Face-to-face interviews were conducted to collect data from all participants by trained interviewers, and anthropometric measurements were performed by trained nurses. All participants or their legal proxies signed the informed consent, and the Research Ethics Committee of Sichuan University approved the study.

### Measurement of laboratory parameters

Venous blood samples were collected in the morning after an overnight fast to measure the laboratory parameters including platelet counts, neutrophil counts, lymphocyte counts, monocyte counts, hemoglobin, fasting plasma glucose, plasma lipids, total bilirubin, direct bilirubin, total protein, albumin, alanine aminotransferase (ALT), aspartate aminotransferase (AST), γ-glutamyl transpeptidase (GGT), alkaline phosphatase, Cystatin C, creatinine, and uric acid using standard kits (Roche Diagnostics, Switzerland). The level of globulin was obtained indirectly by subtracting the level of albumin from the level of total protein. Additionally, CRP levels were detected with the Modular Analytics Cobas® 6000 (Roche Diagnostics, Switzerland) by an immunoturbidimetric technique.

### Calculation of the inflammatory indexes

PLR and NLR were calculated as the absolute platelet counts and neutrophil counts divided by the absolute lymphocyte counts, respectively. LMR was calculated as the absolute lymphocyte counts divided by the absolute monocyte counts.

### Measurement of muscle mass, muscle strength, and physical performance

According to the recommendation of the Asia Working Group for Sarcopenia (AWGS) [17], a bioimpedance analysis (BIA) device (InBody 230; Biospace Co. Ltd., Seoul, Korea) was used to estimate the appendicular skeletal muscle mass (ASM) and body fat mass. Then, the appendicular skeletal muscle mass index (ASMI) was calculated by the equation: ASMI (kg/m^2^) = ASM/height^2^. Handgrip strength was measured using a handheld dynamometer (EH101, Xiangshan Inc., Guangdong, China) [17]. After three measurements of the handgrip strength of both hands, the best result of either hand was used for the analyses. Gait speed was measured as the time consumed to walk a 4-m course at usual speed to estimate physical performance [17, 18]. Canes or walkers were allowed during the walking test, if necessary.

### Assessment of sarcopenia

We defined sarcopenia according to different criteria: the AWGS [17], the updated version of AWGS (AWGS 2019) [19], the European Working Group on Sarcopenia in Older People (EWGSOP) [20], the updated version of EWGSOP (EWGSOP2) [2], the International Working Group on Sarcopenia (IWGS) [21], and the Foundation for the National Institutes of Health Sarcopenia Project (FNIH) [22]. The detailed criteria used in this study are listed in Supplementary Table 1.

### Assessment of potential covariates

Covariates such as gender, age, and comorbidities (hypertension, coronary heart disease, diabetes, stroke, chronic obstructive pulmonary disease, cognitive impairment, and history of falls in the previous year) were collected from the face-to-face interviews. The participants’ body weight and height were measured by trained nurses. The body mass index (BMI) was then calculated by the equation: BMI (kg/m^2^) = body weight/height^2^. The calf circumference (CC) was measured around the left calf at the widest part, with the participants lie in a supine position and left knee bent at a right angle, by a millimeter graded tape.

### Statistical analysis

For categorical variables, the data were presented as numbers (percentages), and the chi-squared tests were used to compare the differences between groups. The continuous data were of normal distribution according to the Shapiro-Wilk test. Therefore, for the continuous variables, the data were presented as mean ± standard deviation (SD), and the differences between groups were compared using the one-way ANOVA test.

Pearson’s r was calculated to detect the correlations between the inflammatory indexes and the component of sarcopenia (ASM, gait speed, and handgrip strength) and CRP. Moreover, multiple logistic regression analysis was performed to calculate the adjusted odds ratios (ORs) and 95% confidence intervals (CIs) for sarcopenia by the inflammatory indexes and CRP. The inflammatory indexes and CRP were treated both as continuous variables (per 1-SD) and as categorical variables (using quartile cutoff points). Model 1 was adjusted for age and gender. Model 2 further adjusted for coronary heart disease and cognitive impairment. Model 3 included Model 2 + albumin, high-density lipoprotein cholesterol (HDL-C), and BMI.

Data analyses were performed using SPSS version 20.0 (SPSS Inc., Chicago, IL, USA). *P* < 0.05 was considered statistically significant.

## Results

### Baseline characteristics of the study population

We included 384 participants (224 women and 160 men) with a mean age of 71.5 ± 5.8 years. Table 1 shows the characteristics of our study population according to AWGS-defined sarcopenia. Participants with sarcopenia were significantly older than those without sarcopenia (*p* < 0.001). Compared with the non-sarcopenia group, the sarcopenia group was more likely to have coronary heart disease (18.0% vs. 7.7%, *p* = 0.011), cognitive impairment (13.1% vs. 1.9%, p < 0.001), and a history of falls in the previous year (26.2% vs. 13.3%, *p* = 0.010). The level of globulin and HDL-C in the sarcopenia group were significantly higher than those in the non-sarcopenia group, whereas the level of albumin and hemoglobin were significantly lower in the sarcopenia group. Very similar results were found when the study population was grouped according to AWGS 2019-defined sarcopenia (Supplementary Table 2).

**Table 1:** Baseline characteristics of participants according to AWGS-defined sarcopenia

Characteristics	No sarcopenia (***n*** = 323)	Sarcopenia (***n*** = 61)	***p***
Women (%)	182 (56.3)	42 (68.9)	0.069
Age (years)	70.7 ± 5.3	75.9 ± 6.5	< 0.001
Comorbidities (%)			
Hypertension	96 (29.7)	20 (32.8)	0.632
Coronary heart disease	25 (7.7)	11 (18.0)	0.011
Diabetes	29 (9.0)	7 (11.5)	0.539
Stoke	41 (12.7)	6 (9.8)	0.532
COPD	28 (8.7)	4 (6.6)	0.584
Cognitive impairment	6 (1.9)	8 (13.1)	< 0.001
History of falls	43 (13.3)	16 (26.2)	0.010
BMI (kg/m^2^)	24.4 ± 3.4	23.4 ± 2.9	0.025
CC (cm)	32.7 ± 2.6	30.8 ± 2.2	< 0.001
ASM (kg)	15.4 ± 3.8	12.3 ± 2.6	< 0.001
ASMI (kg/m^2^)	6.3 ± 1.0	5.4 ± 0.8	< 0.001
Body fat mass (kg)	19.0 ± 5.8	18.5 ± 5.1	0.531
Gait speed (m/s)	0.9 ± 0.2	0.6 ± 0.1	< 0.001
Handgrip strength (kg)	24.4 ± 8.7	14.8 ± 4.8	< 0.001
Laboratory parameters			
Total bilirubin (μmol/L)	15.2 ± 6.4	15.2 ± 6.3	0.956
Direct bilirubin (μmol/L)	5.1 ± 1.8	4.9 ± 1.7	0.388
ALT (IU/L)	19.3 ± 10.2	20.0 ± 17.3	0.623
AST (IU/L)	23.0 ± 7.0	24.9 ± 13.4	0.115
Albumin (g/L)	43.1 ± 2.6	42.2 ± 2.9	0.007
Globulin (g/L)	28.6 ± 4.2	30.2 ± 4.7	0.007
Alkaline phosphatase (IU/L)	78.7 ± 21.0	81.9 ± 23.7	0.285
GGT (IU/L)	23.5 ± 15.9	24.3 ± 21.6	0.714
Creatinine (μmol/L)	74.3 ± 17.8	73.4 ± 20.8	0.732
Cystatin C (mg/L)	1.0 ± 0.2	1.1 ± 0.2	0.230
Uric acid (μmol/L)	332.5 ± 82.7	311.0 ± 81.6	0.063
Glucose (mmol/L)	5.5 ± 1.4	5.6 ± 1.8	0.442
Triglyceride (mmol/L)	1.5 ± 1.0	1.3 ± 0.7	0.226
Total cholesterol (mmol/L)	4.5 ± 0.9	4.6 ± 1.0	0.481
HDL-C (mmol/L)	1.4 ± 0.4	1.6 ± 0.4	0.004
LDL-C (mmol/L)	2.9 ± 0.8	2.9 ± 0.9	0.955
CRP (mg/L)	2.7 ± 1.9	2.9 ± 2.0	0.420
Hemoglobin (g/L)	137.2 ± 15.6	129.2 ± 16.1	< 0.001
Platelet (10^9/L)	148.8 ± 52.3	157.6 ± 59.7	0.240
Leukocyte (10^9/L)	5.5 ± 1.4	5.4 ± 1.6	0.488
Neutrophil (10^9/L)	3.3 ± 1.0	3.2 ± 1.2	0.638
Lymphocyte (10^9/L)	1.7 ± 0.5	1.6 ± 0.5	0.482
Monocyte (10^9/L)	0.4 ± 0.2	0.4 ± 0.1	0.406

Notes**:** Data are presented as the number (percentage) for the following variables: women and specific comorbidities listed above. For other variables, the mean ± SD is applied

One-way ANOVA and chi-squared tests were used where appropriate. P < 0.05 indicates statistically significant

*Abbreviations*: *ALT* alanine aminotransferase, *ASM* appendicular skeletal muscle mass, *ASMI* appendicular skeletal muscle index, *AST* aspartate aminotransferase, *BMI* body mass index, *CC* calf circumference, *COPD* chronic obstructive pulmonary disease, *CRP* C-reactive protein, *GGT* γ-glutamyl transpeptidase, *HDL-C* high-density lipoprotein cholesterol, *LDL-C* low-density lipoprotein cholesterol

There were no significant differences concerning the PLR, NLR, and LMR between the sarcopenia group and the non-sarcopenia group regardless of the diagnostic criteria of sarcopenia (Table 2).

**Table 2:** Group difference of the inflammatory indexes according to sarcopenia defined by different criteria

Characteristics	No sarcopenia	Sarcopenia	***p***
**AWGS**	*n* = 323	*n* = 61	
PLR	94.3 ± 39.5	101.6 ± 40.6	0.190
NLR	2.1 ± 0.9	2.1 ± 0.8	0.828
LMR	4.4 ± 1.3	4.4 ± 1.5	0.939
**AWGS 2019**	*n* = 208	*n* = 176	
PLR	93.7 ± 35.5	97.5 ± 44.2	0.348
NLR	2.1 ± 0.8	2.1 ± 1.0	0.407
LMR	4.4 ± 1.5	4.4 ± 1.6	0.757
**EWGSOP**	*n* = 339	*n* = 45	
PLR	94.2 ± 39.6	105.3 ± 39.4	0.079
NLR	2.1 ± 0.9	2.3 ± 1.2	0.078
LMR	4.5 ± 1.5	4.1 ± 1.5	0.141
**EWGSOP2**	*n* = 346	*n* = 38	
PLR	94.5 ± 39.7	104.3 ± 38.9	0.149
NLR	2.1 ± 0.9	2.3 ± 1.1	0.177
LMR	4.4 ± 1.5	4.4 ± 1.6	0.909
**IWGS**	*n* = 288	*n* = 96	
PLR	94.1 ± 38.6	99.7 ± 42.9	0.228
NLR	2.1 ± 0.9	2.1 ± 0.9	0.758
LMR	4.4 ± 1.5	4.4 ± 1.5	0.884
**FNIH**	*n* = 325	*n* = 59	
PLR	96.1 ± 40.2	92.1 ± 37.1	0.484
NLR	2.1 ± 0.9	1.9 ± 0.8	0.178
LMR	4.4 ± 1.5	4.7 ± 1.4	0.078

*Abbreviations*: *AWGS* Asia Working Group for Sarcopenia, *AWGS 2019* the updated version of AWGS, *EWGSOP* European Working Group on Sarcopenia in Older People, *EWGSOP2* the updated version of EWGSOP, *FNIH* the Foundation for the National Institutes of Health, *IWGS* International Working Group on Sarcopenia, *LMR* lymphocyte-to-monocyte ratio, *NLR* neutrophil-to-lymphocyte ratio, *PLR* platelet-to-lymphocyte ratio

### Prevalence of sarcopenia

Among the 384 participants, between 38 and 176 subjects were diagnosed with sarcopenia depending on the diagnostic criteria applied. The prevalence of sarcopenia thus varied from 9.9% (EWGSOP2) to 45.8% (AWGS 2019). According to the AWGS criteria, 61 participants (15.9%) had sarcopenia. The prevalence of sarcopenia was not significantly different in men and women (11.9% vs. 18.8%, respectively; *P* = 0.069).

### Correlation between the inflammatory indexes and ASM, gait speed, handgrip strength, and CRP

As shown in Table 3, PLR was significantly but slightly correlated with ASM (r = − 0.103, *p* = 0.044), while LMR was significantly but slightly correlated with handgrip strength (r = − 0.121, *p* = 0.018).

**Table 3:** Correlations between the inflammatory indexes and ASM, gait speed, handgrip strength, and CRP

	ASM		Gait speed		Handgrip strength		CRP	
	r	P	r	p	r	p	r	p
PLR	−0.103	0.044^*^	−0.026	0.611	−0.076	0.135	0.004	0.942
NLR	−0.018	0.726	0.052	0.308	0.014	0.787	0.029	0.572
LMR	−0.100	0.051	−0.072	0.162	−0.121	0.018^*^	−0.050	0.330

Notes: *Significant at 0.05 level

*Abbreviations*: *ASM* appendicular skeletal muscle mass, *CRP* C-reactive protein, *LMR* lymphocyte-to-monocyte ratio, *NLR* neutrophil-to-lymphocyte ratio, *PLR* platelet-to-lymphocyte ratio, *r* Pearson’s r

### Association between the inflammatory indexes, CRP, and sarcopenia

As shown in Table 4, PLR, NLR, LMR, or CRP was not significantly associated with AWGS-defined sarcopenia (PLR per 1-SD: OR 1.19, 95% CI 0.92 to 1.53; NLR per 1-SD: OR 0.97, 95% CI 0.73 to 1.28; LMR per 1-SD: OR 0.99, 95% CI 0.75 to 1.30; CRP per 1-SD: OR 1.11, 95% CI 0.86 to 1.44). After fully adjusting for multiple covariates, similar results were identified (PLR per 1-SD: adjusted OR 1.09, 95% CI 0.82 to 1.45; NLR per 1-SD: adjusted OR 0.96, 95% CI 0.71 to 1.30; LMR per 1-SD: adjusted OR 1.01, 95% CI 0.74 to 1.38; CRP per 1-SD: adjusted OR 1.14, 95% CI 0.85 to 1.54).

**Table 4:** Association between PLR, NLR, LMR, CRP (per 1-SD) and sarcopenia according to Logistic Regression Models adjusted for potential confounders

	Unadjusted	Model 1	Model 2	Model 3
**AWGS-defined sarcopenia**				
PLR (per 1-SD)	1.19 (0.92–1.53)	1.16 (0.88–1.53)	1.19 (0.91–1.58)	1.09 (0.82–1.45)
NLR (per 1-SD)	0.97 (0.73–1.28)	1.04 (0.77–1.40)	1.04 (0.77–1.40)	0.96 (0.71–1.30)
LMR (per 1-SD)	0.99 (0.75–1.30)	0.97 (0.72–1.32)	0.96 (0.70–1.31)	1.01 (0.74–1.38)
CRP (per 1-SD)	1.11 (0.86–1.44)	1.07 (0.81–1.42)	1.06 (0.80–1.41)	1.14 (0.85–1.54)
**AWGS 2019-defined sarcopenia**				
PLR (per 1-SD)	1.10 (0.90–1.35)	1.09 (0.89–1.35)	1.10 (0.89–1.36)	0.86 (0.67–1.10)
NLR (per 1-SD)	1.09 (0.89–1.33)	1.11 (0.90–1.37)	1.10 (0.89–1.36)	0.91 (0.71–1.17)
LMR (per 1-SD)	0.97 (0.79–1.19)	1.00 (0.81–1.25)	1.01 (0.82–1.26)	1.15 (0.89–1.47)
CRP (per 1-SD)	0.91 (0.75–1.12)	0.89 (0.71–1.10)	0.89 (0.71–1.10)	1.10 (0.86–1.41)
**EWGSOP-defined sarcopenia**				
PLR (per 1-SD)	1.28 (0.97–1.69)	1.27 (0.95–1.70)	1.27 (0.95–1.70)	1.01 (0.74–1.38)
NLR (per 1-SD)	1.28 (0.97–1.68)	1.36 (1.02–1.81)	1.37 (1.02–1.82)	1.14 (0.83–1.56)
LMR (per 1-SD)	0.78 (0.56–1.09)	0.77 (0.54–1.10)	0.75 (0.53–1.08)	0.86 (0.58–1.27)
CRP (per 1-SD)	0.81 (0.56–1.18)	0.17 (0.75–1.13)	0.74 (0.49–1.11)	0.93 (0.61–1.43)
**EWGSOP2-defined sarcopenia**				
PLR (per 1-SD)	1.25 (0.92–1.68)	1.24 (0.91–1.69)	1.23 (0.89–1.69)	0.99 (0.71–1.38)
NLR (per 1-SD)	1.23 (0.91–1.65)	1.28 (0.94–1.75)	1.30 (0.95–1.77)	1.11 (0.79–1.54)
LMR (per 1-SD)	1.02 (0.73–1.42)	1.07 (0.75–1.53)	1.05 (0.73–1.51)	1.26 (0.84–1.87)
CRP (per 1-SD)	1.00 (0.72–1.40)	0.97 (0.68–1.38)	0.95 (0.66–1.36)	1.17 (0.79–1.74)
**IWGS-defined sarcopenia**				
PLR (per 1-SD)	1.15 (0.92–1.43)	1.14 (0.90–1.44)	1.13 (0.89–1.44)	1.04 (0.81–1.33)
NLR (per 1-SD)	0.96 (0.76–1.22)	0.99 (0.77–1.27)	0.98 (0.77–1.26)	0.92 (0.71–1.18)
LMR (per 1-SD)	0.98 (0.78–1.24)	1.02 (0.79–1.32)	1.02 (0.79–1.32)	1.07 (0.83–1.39)
CRP (per 1-SD)	0.96 (0.76–1.22)	0.92 (0.78–1.19)	0.92 (0.71–1.19)	0.98 (0.75–1.28)
**FNIH-defined sarcopenia**				
PLR (per 1-SD)	0.90 (0.67–1.21)	0.85 (0.62–1.17)	0.85 (0.61–1.17)	0.88 (0.63–1.23)
NLR (per 1-SD)	0.81 (0.59–1.10)	0.82 (0.59–1.15)	0.83 (0.59–1.16)	0.85 (0.60–1.20)
LMR (per 1-SD)	1.27 (0.97–1.66)	1.42 (1.04–1.93)	1.40 (1.03–1.91)	1.39 (1.01–1.90)
CRP (per 1-SD)	1.09 (0.84–1.42)	1.05 (0.78–1.41)	1.04 (0.77–1.40)	1.02 (0.74–1.40)

**Notes:** Data are presented as odds ratios (95% confidential intervals). PLR, NLR, MLR were treated as continuous variables (per 1-SD)

Model 1: adjusted for age and gender. Model 2: adjusted for age, gender, coronary heart disease, and cognitive impairment. Model 3: adjusted for age, gender, coronary heart disease, cognitive impairment, albumin, HDL-C, and BMI

***Abbreviations*:** *AWGS* Asia Working Group for Sarcopenia, *AWGS 2019* the updated version of AWGS, *CRP* C-reactive protein, *EWGSOP* European Working Group on Sarcopenia in Older People, *EWGSOP2* the updated version of EWGSOP, *FNIH* the Foundation for the National Institutes of Health, *IWGS* International Working Group on Sarcopenia, *LMR* lymphocyte-to-monocyte ratio, *NLR* neutrophil-to-lymphocyte ratio, *PLR* platelet-to-lymphocyte ratio, *SD* standard deviation

Moreover, similar results were found when sarcopenia was defined by the AWGS 2019, the EWGSOP, the EWGSOP2, the IWGS, or the FNIH, respectively (Table 4), and similar results were found when PLR, NLR, LMR, CRP were treated as categorical variables (using quartile cutoff points) regardless of the diagnostic criteria (Supplementary Table 3, 4, 5, 6, 7 and 8).

## Discussion

This study showed that none of the three inflammatory indexes (PLR, NLR, or LMR) were significantly associated with sarcopenia in our study population regardless of the diagnostic criteria of sarcopenia. Additionally, no significant correlation between the three inflammatory indexes (PLR, NLR, or LMR) and serum CRP levels was found. However, PLR and LMR were significantly but slightly correlated with ASM and handgrip strength, respectively.

Our finding that the inflammatory indexes were not correlated with CRP levels and not associated with sarcopenia was not in line with previous studies. For example, a cross-sectional study conducted in the outpatient clinic found that NLR values were positively correlated with other inflammatory markers, such as CRP; and a higher NLR level was independently associated with an increased risk of EWGSOP defined sarcopenia [14]. The discrepancy may be related to the different settings of studies. In another cross-sectional study enrolled 3671 community-dwelling older adults, the PLR values were also positively correlated with CRP, and the elevations in PLR values were positively associated with sarcopenia status [15]. However, the determination of sarcopenia status was only based on the bioelectrical impedance analysis equation of Janssen et al. [23], which cannot reflect other important components of sarcopenia, such as muscle strength and physical performance. Based on present evidence, whether the inflammatory indexes can represent the inflammation in sarcopenia or be used as indicators of sarcopenia remains controversial.

In addition, our finding that CRP levels were not significantly associated with sarcopenia in community-dwelling older people was not in line with previous studies. For example, pooling data from 16 studies showed that sarcopenia was associated with a higher level of CRP [8]. The discrepancy may result from the different characteristics of the participants, those who included in these studies were more likely to have a greater degree of inflammation-driven by the underlying diseases, such as ankylosing spondylitis [24], ulcerative colitis [25], chronic obstructive pulmonary disease [26], end-stage renal disease [27], and malignancy disease [28–30].

Interestingly, we found that PLR and LMR were significantly but slightly correlated with ASM and handgrip strength respectively in the same study population. This finding was similar to previous studies. For example, in a cross-sectional study conducted in community-dwelling older adults, the elevations in PLR values were negatively associated with ASMI [15]. Meanwhile, a strong negative correlation was identified between free fat mass and NLR levels in sarcopenia patients admitted to the outpatient clinics [14].

The inflammatory indexes have been widely studied in patients with malignant diseases [12] and neurodegenerative diseases [13]. Some studies demonstrated that the inflammatory indexes were associated with other inflammatory indicators, such as IL-6, CRP, and TNF-α, and were useful for predicting disease progression, treatment response, and prognosis [12, 31]. These findings imply that the inflammatory indexes may reflect the degree of inflammation in these diseases. However, other studies found the opposite results. For example, a prospective study conducted in 317 patients with muscle-invasive bladder cancer suggested that NLR was neither a prognostic nor a predictive biomarker for overall survival [32]. One explanation for the discrepancy of these results may be the reporting and publication bias. For example, in a meta-analysis about the correlation of NLR with outcomes of urothelial carcinoma, publication bias could not be excluded by inverted funnel plots [12]. An analysis of 1915 publications on cancer prognostic markers found that almost all articles reported statistically significant results [33]. Under strong reporting bias, the statistical significance may lose the ability to distinguish the importance of prognostic or predictive markers [33].

Some intrinsic defects of the inflammatory indexes may also limit their utility as biomarkers of various diseases. First, the mechanisms of how the inflammatory indexes predict disease prognosis remain unknown. As reported by Seok-Jin Choi, a high NLR indicated short survival duration in patients with amyotrophic lateral sclerosis (ALS), but there was no significant correlation between the NLR and CRP levels [13]. They suggested that a high NLR in ALS may not be a consequence of systemic inflammation, but may be due to the specific immune modulations underlying the pathogenesis of ALS. Second, it is difficult to establish optimal cut-off values for the inflammatory indexes. The current cut-off values widely vary across studies [34, 35]. For example, Gary et al. identified that a PLR of 150 could serve as a cut-off value in patients with limb ischemia caused by peripheral arterial occlusive disease [36]. Another study suggested that a PLR of 116.85 for identifying sarcopenia in operable gastric cancer patients [37]. Moreover, the reported cut-off values for NLR as a poor prognostic indicator in patients with solid tumors varied from 1.9 to 7.2 [34].

Our study has some limitations. First, this was a cross-sectional study conducted at a single institution. Second, the sample size of our study is relatively small to draw a robust conclusion. Third, we did not adjust for some important possible confounders, such as activities of daily living and frailty. Last, the calculation of these inflammatory indexes was dependent on a single measurement, therefore the accuracy of results could be affected by potential laboratory measurement errors and biologic variabilities.

## Conclusions

In Chinese community-dwelling older people, the inflammatory indexes (PLR, NLR, and LMR) were not significantly associated with sarcopenia, although PLR and LMR were significantly but slightly associated with some components of sarcopenia (ASM and handgrip strength), respectively. Our study did not support these inflammatory indexes as the biomarkers for sarcopenia in Chinese community-dwelling older people. However, considering the inflammatory indexes can be simply calculated from a routine blood test without adding any burden to patients or medical staffs, further studies remain warranted to validate the association between the inflammatory indexes and sarcopenia in different populations, especially among those who are supposed to have low-grade chronic inflammation, such as the oldest old and the patients with diabetes, chronic obstructive pulmonary disease, or cancer.

## Supplementary Information

**Additional file 1 Table S1.** The diagnostic criteria for sarcopenia used in this study.

**Additional file 2 Table S2.** Baseline characteristics of participants according to AWGS 2019-defined sarcopenia.

**Additional file 3 Table S3.** Association between PLR, NLR, LMR, CRP, and AWGS-defined sarcopenia according to Logistic Regression Models adjusted for potential confounders.

**Additional file 4 Table S4.** Association between PLR, NLR, LMR, CRP, and AWGS 2019-defined sarcopenia according to Logistic Regression Models adjusted for potential confounder.

**Additional file 5 Table S5.** Association between PLR, NLR, LMR, CRP, and EWGSOP-defined sarcopenia according to Logistic Regression Models adjusted for potential confounders.

**Additional file 6 Table S6.** Association between PLR, NLR, LMR, CRP, and EWGSOP2-defined sarcopenia according to Logistic Regression Models adjusted for potential confounders.

**Additional file 7 Table S7.** Association between PLR, NLR, LMR, CRP, and IWGS-defined sarcopenia according to Logistic Regression Models adjusted for potential confounders.

**Additional file 8 Table S8.** Association between PLR, NLR, LMR, CRP, and FNIH-defined sarcopenia according to Logistic Regression Models adjusted for potential confounders.

## Data Availability

Data that support the findings of this study are available from the corresponding author on reasonable request.

## Abbreviations

ALT: Alanine aminotransferase
ASM: Appendicular skeletal muscle mass
ASMI: Appendicular skeletal muscle mass index
AST: Aspartate aminotransferase
AWGS: Asian Working Group for Sarcopenia
AWGS 2019: The updated version of AWGS
BIA: Bioimpedance analysis
BMI: Body mass index
CC: Calf circumference
CI: Confidence intervals
CRP: C-reactive protein
EWGSOP: European Working Group on Sarcopenia in Older People
EWGSOP2: The updated version of EWGSOP
FNIH: Foundation for the National Institutes of Health Sarcopenia Project
GGT: γ-glutamyl transpeptidase
HDL-C: High-density lipoprotein cholesterol
ICD-10-CM: International classification of disease, tenth revision, clinical modification
IL-6: Interleukin- 6
IWGS: International Working Group on Sarcopenia
LMR: Lymphocyte-to-monocyte ratio
NLR: Neutrophil-to-lymphocyte ratio
OR: Odds ratio
PLR: Platelet-to-lymphocyte ratio
SD: Standard deviation
TNF-α: Tumor necrosis factor α
