# Supplementary material for: Inflammatory indexes are not associated with sarcopenia in Chinese community-dwelling older people: a cross-sectional study
Source: BMC Geriatr. 2020 Nov 7;20:457. doi: 10.1186/s12877-020-01857-5 (PMC7648963; doi:10.1186/s12877-020-01857-5)
Supplement: Supplementary file 1 — Additional file 1 Table S1. The diagnostic criteria for sarcopenia used in this study. [file 12877_2020_1857_MOESM1_ESM.docx]

**Supplementary Table 1. The diagnostic criteria for sarcopenia used in this study**

|  | **① Low muscle mass** | **② Low handgrip strength (kg)** | **③ Low gait speed (m/s)** | **Diagnostic criteria** |
| --- | --- | --- | --- | --- |
| EWGSOP | ASMI ≤7.26 kg/m^2^ for men;  ASMI ≤5.50 kg/m^2^ for women | <30 for men;  <20 for women | <0.8 for both gender | ① + ② or ① + ③ |
| EWGSOP2 | ASMI ≤7.0 kg/m^2^ for men;  ASMI ≤6.0 kg/m^2^ for women | <27 for men;  <16 for women | ≤0.8 for both gender | ① + ② or ① + ② + ③ |
| IWGS | ASMI ≤7.23 kg/m^2^ for men;  ASMI ≤5.67 kg/m^2^ for women | Not applicable | <1.0 for both gender | ① + ③ |
| FNIH | ALM_BMI_ <0.789 for men  ALM_BMI_ <0.512 for women | <26 for men;  <16 for women | Not applicable | ① + ② |
| AWGS | ASMI <7.0 kg/m^2^ for men;  ASMI <5.7 kg/m^2^ for women | <26 for men;  <18 for women | <0.8 for both gender | ①+ ② or ① + ③ |
| AWGS 2019 | ASMI <7.0 kg/m^2^ for men;  ASMI <5.7 kg/m^2^ for women | <28 for men;  <18 for women | <1.0 for both gender | ①+ ② or ① + ③ |

**Abbreviations:** ALM, appendicular skeletal lean mass; ASMI, appendicular skeletal muscle mass index; AWGS, Asia Working Group for Sarcopenia; AWGS 2019, the updated version of AWGS; BMI, body mass index; EWGSOP, European Working Group on Sarcopenia in Older People; EWGSOP2, the updated version of EWGSOP; FNIH, Foundation for the National Institutes of Health; IWGS, International Working Group on Sarcopenia.
