# Supplementary material for: Inflammatory indexes are not associated with sarcopenia in Chinese community-dwelling older people: a cross-sectional study
Source: BMC Geriatr. 2020 Nov 7;20:457. doi: 10.1186/s12877-020-01857-5 (PMC7648963; doi:10.1186/s12877-020-01857-5)
Supplement: Supplementary file 2 — Additional file 2 Table S2. Baseline characteristics of participants according to AWGS 2019-defined sarcopenia. [file 12877_2020_1857_MOESM2_ESM.docx]

**Supplementary Table 2. Baseline characteristics of participants according to AWGS 2019-defined sarcopenia**

| **Characteristics** | **No sarcopenia**  **(n=208)** | **Sarcopenia**  **(n=176)** | **p** |
| --- | --- | --- | --- |
| Women (%) | 121 (58.2) | 103 (58.5) | 0.945 |
| Age (years) | 70.0 ± 5.0 | 73.3 ± 6.2 | <0.001 |
| Comorbidities (%) | | | |
| Hypertension | 65 (31.3) | 51 (29.0) | 0.629 |
| Coronary heart disease | 14 (6.7) | 22 (12.5) | 0.053 |
| Diabetes | 21 (10.1) | 15 (8.5) | 0.598 |
| Stoke | 28 (13.5) | 19 (10.8) | 0.427 |
| COPD | 21 (10.1) | 11 (6.3) | 0.174 |
| Cognitive impairment | 4 (1.9) | 10 (5.7) | 0.050 |
| History of falls | 25 (12.0) | 34 (19.3) | 0.048 |
| BMI (kg/m^2^) | 25.6 ± 3.1 | 22.6 ± 2.8 | <0.001 |
| CC (cm) | 33.6± 2.4 | 31.0 ± 2.1 | <0.001 |
| ASM (kg) | 16.4 ± 3.8 | 13.1 ± 2.8 | <0.001 |
| ASMI (kg/m^2^) | 6.6 ± 1.0 | 5.6 ± 0.8 | <0.001 |
| Body fat mass (kg) | 20.7 ± 5.6 | 16.9 ± 5.0 | <0.001 |
| Gait speed (m/s) | 1.0 ± 0.2 | 0.8 ± 0.1 | <0.001 |
| Handgrip strength (kg) | 25.7 ± 9.2 | 19.5 ± 7.2 | <0.001 |
| Laboratory parameters |  |  |  |
| Total bilirubin (μmol/L) | 15.2 ± 5.9 | 15.3 ± 6.9 | 0.909 |
| Direct bilirubin (μmol/L) | 5.0 ± 1.7 | 5.1 ± 1.8 | 0.509 |
| ALT (IU/L) | 19.9 ± 10.8 | 18.7 ± 12.5 | 0.322 |
| AST (IU/L) | 22.8 ± 6.9 | 23.9 ± 9.8 | 0.236 |
| Albumin (g/L) | 43.4 ± 2.6 | 42.5 ± 2.6 | 0.002 |
| Globulin (g/L) | 28.5 ± 4.2 | 29.4 ± 4.4 | 0.039 |
| Alkaline phosphatase (IU/L) | 79.5 ± 21.8 | 78.8 ± 21.1 | 0.746 |
| GGT (IU/L) | 24.6 ± 18.1 | 22.5 ± 15.3 | 0.217 |
| Creatinine (μmol/L) | 74.8 ± 17.9 | 73.3 ± 18.7 | 0.430 |
| Cystatin C (mg/L) | 1.0 ± 0.2 | 1.0 ± 0.2 | 0.487 |
| Uric acid (μmol/L) | 343.7 ± 83.7 | 311.7 ± 78.4 | <0.001 |
| Glucose (mmol/L) | 5.5 ± 1.4 | 5.5 ± 1.6 | 0.655 |
| Triglyceride (mmol/L) | 1.7 ± 1.1 | 1.3 ± 0.6 | <0.001 |
| Total cholesterol (mmol/L) | 4.5 ± 0.9 | 4.5 ± 0.9 | 0.982 |
| HDL-C (mmol/L) | 1.3 ± 0.3 | 1.5 ± 0.4 | <0.001 |
| LDL-C (mmol/L) | 2.9 ± 0.8 | 2.9 ± 0.8 | 0.413 |
| CRP (mg/L) | 2.8 ± 2.1 | 2.7 ± 1.8 | 0.390 |
| Hemoglobin (g/L) | 137.8 ± 15.5 | 133.8 ± 16.2 | 0.012 |
| Platelet (10^9/L) | 149.5 ± 52.9 | 151.0 ± 54.4 | 0.782 |
| Leukocyte (10^9/L) | 5.5 ± 1.4 | 5.5 ± 1.4 | 0.964 |
| Neutrophil (10^9/L) | 3.2 ± 1.1 | 3.2 ± 1.0 | 0.932 |
| Lymphocyte (10^9/L) | 1.7 ± 0.5 | 1.7 ± 0.5 | 0.699 |
| Monocyte (10^9/L) | 0.4 ± 0.2 | 0.4 ± 0.1 | 0.844 |

**Notes:** Data are presented as the number (percentage) for the following variables: women and specific comorbidities listed above. For other variables, the mean ± SD is applied.

One-way ANOVA and chi-squared tests were used where appropriate. P <0.05 indicates statistically significant.

**Abbreviations:** ALT, alanine aminotransferase; ASM, appendicular skeletal muscle mass; ASMI, appendicular skeletal muscle index; AST, aspartate aminotransferase; AWGS 2019, the updated version of Asian Working Group for Sarcopenia; BMI, body mass index; CC, calf circumference; COPD, chronic obstructive pulmonary disease; CRP, C-reactive protein; GGT, γ-glutamyl transpeptidase; HDL-C, high-density lipoprotein cholesterol; LDL-C, low-density lipoprotein cholesterol; LMR, lymphocyte-to-monocyte ratio; NLR, neutrophil-to-lymphocyte ratio; PLR, platelet-to-lymphocyte ratio.
