# Supplementary material for: Inflammatory indexes are not associated with sarcopenia in Chinese community-dwelling older people: a cross-sectional study
Source: BMC Geriatr. 2020 Nov 7;20:457. doi: 10.1186/s12877-020-01857-5 (PMC7648963; doi:10.1186/s12877-020-01857-5)
Supplement: Supplementary file 3 — Additional file 3 Table S3. Association between PLR, NLR, LMR, CRP, and AWGS-defined sarcopenia according to Logistic Regression Models adjusted for potential confounders. [file 12877_2020_1857_MOESM3_ESM.docx]

**Supplementary Table 3. Association between PLR, NLR, LMR, CRP, and AWGS-defined sarcopenia according to Logistic Regression Models adjusted for potential confounders**

|  | **Unadjusted** | **Model 1** | **Model 2** | **Model 3** |
| --- | --- | --- | --- | --- |
| PLR (per 1-SD) | 1.19 (0.92-1.53) | 1.16 (0.88-1.53) | 1.19 (0.91-1.58) | 1.09 (0.82-1.45) |
| Quartile of PLR |  |  |  |  |
| Q1 | 0.79 (0.37-1.72) | 0.78 (0.34-1.80) | 0.72 (0.30-1.68) | 0.84 (0.35-2.02) |
| Q2 | 0.54 (0.23-1.25) | 0.56 (0.23-1.36) | 0.55 (0.23-1.34) | 0.63 (0.26-1.56) |
| Q3 | 1.22 (0.60-2.51) | 1.07 (0.49-2.34) | 1.06 (0.48-2.33) | 1.12 (0.50-2.50) |
| Q4 | 1 (reference) | 1 (reference) | 1 (reference) | 1 (reference) |
| NLR (per 1-SD) | 0.97 (0.73-1.28) | 1.04 (0.77-1.40) | 1. 04 (0.77-1.40) | 0.96 (0.71-1.30) |
| Quartile of NLR |  |  |  |  |
| Q1 | 1.20 (0.58-2.51) | 0.93 (0.42-2.06) | 0.93 (0.41-2.07) | 1.06 (0.46-2.41) |
| Q2 | 0.65 (0.28-1.48) | 0.52 (0.22-1.28) | 0.51 (0.21-1.26) | 0.55 (0.22-1.37) |
| Q3 | 0.90 (0.42-1.95) | 0.81 (0.35-1.85) | 0.80 (0.35-1.84) | 0.85 (0.36-1.98) |
| Q4 | 1 (reference) | 1 (reference) | 1 (reference) | 1 (reference) |
| LMR (per 1-SD) | 0.99 (0.75-1.30) | 0.97 (0.72-1.32) | 0.96 (0.70-1.31) | 1.01 (0.74-1.38) |
| Quartile of LMR |  |  |  |  |
| Q1 | 0.83 (0.40-1.74) | 0.89 (0.40-2.01) | 0.90 (0.40-2.05) | 0.76 (0.33-1.77) |
| Q2 | 0.52 (0.23-1.17) | 0.56 (0.24-1.34) | 0.58 (0.24-1.38) | 0.55 (0.23-1.35) |
| Q3 | 0.70 (0.33-1.47) | 0.67 (0.30-1.48) | 0.67 (0.30-1.50) | 0.62 (0.27-1.42) |
| Q4 | 1 (reference) | 1 (reference) | 1 (reference) | 1 (reference) |
| CRP (per 1-SD) | 1.11 (0.86-1.44) | 1.07 (0.81-1.42) | 1.06 (0.80-1.41) | 1.14 (0.85-1.54) |
| Quartile of CRP |  |  |  |  |
| Q1 | 0.55 (0.23-1.32) | 0.73 (0.29-1.84) | 0.74 (0.29-1.90) | 0.50 (0.18-1.38) |
| Q2 | 0.90 (0.41-1.98) | 0.89 (0.38-2.06) | 0.93 (0.40-2.18) | 0.84 (0.35-2.01) |
| Q3 | 1.70 (0.83-3.52) | 1.28 (0.58-2.80) | 1.43 (0.64-3.22) | 1.45 (0.63-3.32) |
| Q4 | 1 (reference) | 1 (reference) | 1 (reference) | 1 (reference) |

**Notes:** Data are presented as odds ratios (95% confidential intervals). PLR, NLR, LMR, CRP were treated as both categorical variables (using quartile cutoff points) and continuous variables (per 1-SD), separately.

Q stands for PLR, NLR, LMR, CRP: Q1 is the lowest quartile and Q4 is the highest quartile. Cutoffs for PLR are Q1<68.2, Q2 68.2-89.3, Q3 89.3-115.3, Q4>115.3. Cutoffs for NLR are Q1<1.5, Q2 1.5-1.9, Q3 1.9-2.5, Q4>2.5. Cutoffs for LMR are Q1<3.3, Q2 3.3-4.3, Q3 4.3-5.4, Q4>5.4. Cutoffs for CRP are Q1<1.5, Q2 1.5-2.1, Q3 2.1-3.2, Q4>3.2.

Model 1: adjusted for age and gender. Model 2: adjusted for age, gender, coronary heart disease, and cognitive impairment. Model 3: adjusted for age, gender, coronary heart disease, cognitive impairment, albumin, HDL-C, and BMI.

**Abbreviations:** AWGS, Asia Working Group for Sarcopenia; CRP, C-reactive protein; LMR, lymphocyte-to-monocyte ratio; NLR, neutrophil-to-lymphocyte ratio; PLR, platelet-to-lymphocyte ratio; SD, standard deviation.
