# Supplementary material for: Inflammatory indexes are not associated with sarcopenia in Chinese community-dwelling older people: a cross-sectional study
Source: BMC Geriatr. 2020 Nov 7;20:457. doi: 10.1186/s12877-020-01857-5 (PMC7648963; doi:10.1186/s12877-020-01857-5)
Supplement: Supplementary file 4 — Additional file 4 Table S4. Association between PLR, NLR, LMR, CRP, and AWGS 2019-defined sarcopenia according to Logistic Regression Models adjusted for potential confounder. [file 12877_2020_1857_MOESM4_ESM.docx]

**Supplementary Table 4. Association between PLR, NLR, LMR, CRP, and AWGS 2019-defined sarcopenia according to Logistic Regression Models adjusted for potential confounders**

|  | **Unadjusted** | **Model 1** | **Model 2** | **Model 3** |
| --- | --- | --- | --- | --- |
| PLR (per 1-SD) | 1.10 (0.90-1.35) | 1.09 (0.89-1.35) | 1.10 (0.89-1.36) | 0.86 (0.67-1.10) |
| Quartile of PLR |  |  |  |  |
| Q1 | 1.00 (0.57-1.76 | 0.99 (0.55-1.80) | 0.97 (0.53-1.76) | 1.67 (0.83-3.36) |
| Q2 | 0.88 (0.50-1.56) | 0.89 (0.49-1.61) | 0.89 (0.49-1.61) | 1.25 (0.63-2.46) |
| Q3 | 0.96 (0.54-1.69) | 0.89 (0.49-1.62) | 0.91 (0.50-1.65) | 1.16 (0.58-2.33) |
| Q4 | 1 (reference) | 1 (reference) | 1 (reference) | 1 (reference) |
| NLR (per 1-SD) | 1.09 (0.89-1.33) | 1.11 (0.90-1.37) | 1.10 (0.89-1.36) | 0.91 (0.71-1.17) |
| Quartile of NLR |  |  |  |  |
| Q1 | 1.19 (0.67-2.09) | 1.09 (0.60-1.96) | 1.12 (0.62-2.02) | 1.72 (0.87-3.41) |
| Q2 | 0.84 (0.47-1.50) | 0.79 (0.44-1.45) | 0.82 (0.45-1.49) | 1.00 (0.50-2.01) |
| Q3 | 1.09 (0.62-1.92) | 1.06 (0.59-1.91) | 1.06 (0.59-1.91) | 1.48 (0.74-2.95) |
| Q4 | 1 (reference) | 1 (reference) | 1 (reference) | 1 (reference) |
| LMR (per 1-SD) | 0.97 (0.79-1.19) | 1.00 (0.81-1.25) | 1.01 (0.82-1.26) | 1.15 (0.89-1.47) |
| Quartile of LMR |  |  |  |  |
| Q1 | 1.19 (0.67-2.10) | 1.13 (0.61-2.09) | 1.11 (0.60-2.05) | 0.74 (0.37-1.51) |
| Q2 | 0.62 (0.35-1.11) | 0.64 (0.35-1.17) | 0.63 (0.34-1.17) | 0.50 (0.24-1.01) |
| Q3 | 0.89 (0.51-1.56) | 0.87 (0.48-1.56) | 0.86 (0.48-1.55) | 0.69 (0.35-1.37) |
| Q4 | 1 (reference) | 1 (reference) | 1 (reference) | 1 (reference) |
| CRP (per 1-SD) | 0.91 (0.75-1.12) | 0.89 (0.71-1.10) | 0.89 (0.71-1.10) | 1.10 (0.86-1.41) |
| Quartile of CRP |  |  |  |  |
| Q1 | 1.14 (0.65-2.02) | 1.32 (0.72-2.41) | 1.33 (0.73-2.43) | 0.52 (0.25-1.09) |
| Q2 | 0.97 (0.54-1.71) | 0.96 (0.53-1.74) | 0.95 (0.52-1.73) | 0.64 (0.31-1.30) |
| Q3 | 1.66 (0.94-2.95) | 1.39 (0.76-2.55) | 1.44 (0.78-2.64) | 1.68 (0.82-3.46) |
| Q4 | 1 (reference) | 1 (reference) | 1 (reference) | 1 (reference) |

**Notes:** Data are presented as odds ratios (95% confidential intervals). PLR, NLR, LMR, CRP were treated as both categorical variables (using quartile cutoff points) and continuous variables (per 1-SD), separately.

Q stands for PLR, NLR, LMR, CRP: Q1 is the lowest quartile and Q4 is the highest quartile. Cutoffs for PLR are Q1<68.2, Q2 68.2-89.3, Q3 89.3-115.3, Q4>115.3. Cutoffs for NLR are Q1<1.5, Q2 1.5-1.9, Q3 1.9-2.5, Q4>2.5. Cutoffs for LMR are Q1<3.3, Q2 3.3-4.3, Q3 4.3-5.4, Q4>5.4. Cutoffs for CRP are Q1<1.5, Q2 1.5-2.1, Q3 2.1-3.2, Q4>3.2.

Model 1: adjusted for age and gender. Model 2: adjusted for age, gender, coronary heart disease, and cognitive impairment. Model 3: adjusted for age, gender, coronary heart disease, cognitive impairment, albumin, HDL-C, and BMI.

**Abbreviations:** AWGS 2019, the updated version of Asia Working Group for Sarcopenia; CRP, C-reactive protein; LMR, lymphocyte-to-monocyte ratio; NLR, neutrophil-to-lymphocyte ratio; PLR, platelet-to-lymphocyte ratio; SD, standard deviation.
