# Supplementary material for: Inflammatory indexes are not associated with sarcopenia in Chinese community-dwelling older people: a cross-sectional study
Source: BMC Geriatr. 2020 Nov 7;20:457. doi: 10.1186/s12877-020-01857-5 (PMC7648963; doi:10.1186/s12877-020-01857-5)
Supplement: Supplementary file 5 — Additional file 5 Table S5. Association between PLR, NLR, LMR, CRP, and EWGSOP-defined sarcopenia according to Logistic Regression Models adjusted for potential confounders. [file 12877_2020_1857_MOESM5_ESM.docx]

**Supplementary Table 5. Association between PLR, NLR, LMR, CRP, and EWGSOP-defined sarcopenia according to Logistic Regression Models adjusted for potential confounders**

|  | **Unadjusted** | **Model 1** | **Model 2** | **Model 3** |
| --- | --- | --- | --- | --- |
| PLR (per 1-SD) | 1.28 (0.97-1.69) | 1.27 (0.95-1.70) | 1.27 (0.95-1.70) | 1.01 (0.74-1.38) |
| Quartile of PLR |  |  |  |  |
| Q1 | 0.49 (0.20-1.22) | 0.47 (0.18-1.20) | 0.48 (0.18-1.24) | 0.73 (0.26-2.08) |
| Q2 | 0.43 (0.17-1.09) | 0.44 (0.17-1.16) | 0.43 (0.16-1.15) | 0.62 (0.21-1.78) |
| Q3 | 1.00 (0.46-2.18) | 0.89 (0.39-2.01) | 0.87 (0.38-1.98) | 1.08 (0.42-2.76) |
| Q4 | 1 (reference) | 1 (reference) | 1 (reference) | 1 (reference) |
| NLR (per 1-SD) | 1.28 (0.97-1.68) | 1.36 (1.02-1.81) | 1.37 (1.02-1.82) | 1.14 (0.83-1.56) |
| Quartile of NLR |  |  |  |  |
| Q1 | 0.74 (0.32-1.72) | 0.59 (0.24-1.44) | 0.57 (0.23-1.40) | 0.81 (0.30-2.18) |
| Q2 | 0.76 (0.33-1.77) | 0.67 (0.28-1.62) | 0.64 (0.26-1.57) | 0.70 (0.26-1.93) |
| Q3 | 0.59 (0.24-1.44) | 0.53 (0.21-1.34) | 0.54 (0.21-1.36) | 0.53 (0.19-1.53) |
| Q4 | 1 (reference) | 1 (reference) | 1 (reference) | 1 (reference) |
| LMR (per 1-SD) | 0.78 (0.56-1.09) | 0.77 (0.54-1.10) | 0.75 (0.53-1.08) | 0.86 (0.58-1.27) |
| Quartile of LMR |  |  |  |  |
| Q1 | 1.41 (0.64-3.10) | 1.51 (0.65-3.51) | 1.54 (0.66-3.60) | 1.07 (0.41-2.84) |
| Q2 | 0.66 (0.27-1.63) | 0.71 (0.28-1.83) | 0.72 (0.28-1.85) | 0.57 (0.20-1.67) |
| Q3 | 0.40 (0.15-1.10) | 0.37 (0.13-1.06) | 0.38 (0.13-1.08) | 0.25 (0.08-0.80) |
| Q4 | 1 (reference) | 1 (reference) | 1 (reference) | 1 (reference) |
| CRP (per 1-SD) | 0.81 (0.56-1.18) | 0.17 (0.75-1.13) | 0.74 (0.49-1.11) | 0.93 (0.61-1.43) |
| Quartile of CRP |  |  |  |  |
| Q1 | 1.61 (0.60-4.34) | 2.12 (0.75-5.96) | 2.20 (0.77-6.31) | 1.04 (0.30-3.52) |
| Q2 | 1.78 (0.67-4.72) | 1.84 (0.67-5.06) | 1.98 (0.71-5.56) | 1.70 (0.53-5.43) |
| Q3 | 2.36 (0.91-6.08) | 1.81 (0.68-4.86) | 1.98 (0.72-5.44) | 2.52 (0.80-7.95) |
| Q4 | 1 (reference) | 1 (reference) | 1 (reference) | 1 (reference) |

**Notes:** Data are presented as odds ratios (95% confidential intervals). PLR, NLR, LMR, CRP were treated as both categorical variables (using quartile cutoff points) and continuous variables (per 1-SD), separately.

Q stands for PLR, NLR, LMR, CRP: Q1 is the lowest quartile and Q4 is the highest quartile. Cutoffs for PLR are Q1<68.2, Q2 68.2-89.3, Q3 89.3-115.3, Q4>115.3. Cutoffs for NLR are Q1<1.5, Q2 1.5-1.9, Q3 1.9-2.5, Q4>2.5. Cutoffs for LMR are Q1<3.3, Q2 3.3-4.3, Q3 4.3-5.4, Q4>5.4. Cutoffs for CRP are Q1<1.5, Q2 1.5-2.1, Q3 2.1-3.2, Q4>3.2.

Model 1: adjusted for age and gender. Model 2: adjusted for age, gender, coronary heart disease, and cognitive impairment. Model 3: adjusted for age, gender, coronary heart disease, cognitive impairment, albumin, HDL-C, and BMI.

**Abbreviations:** CRP, C-reactive protein; EWGSOP, European Working Group on Sarcopenia in Older People; LMR, lymphocyte-to-monocyte ratio; NLR, neutrophil-to-lymphocyte ratio; PLR, platelet-to-lymphocyte ratio; SD, standard deviation.
