# Supplementary material for: Inflammatory indexes are not associated with sarcopenia in Chinese community-dwelling older people: a cross-sectional study
Source: BMC Geriatr. 2020 Nov 7;20:457. doi: 10.1186/s12877-020-01857-5 (PMC7648963; doi:10.1186/s12877-020-01857-5)
Supplement: Supplementary file 6 — Additional file 6 Table S6. Association between PLR, NLR, LMR, CRP, and EWGSOP2-defined sarcopenia according to Logistic Regression Models adjusted for potential confounders. [file 12877_2020_1857_MOESM6_ESM.docx]

**Supplementary Table 6. Association between PLR, NLR, LMR, CRP, and EWGSOP2-defined sarcopenia according to Logistic Regression Models adjusted for potential confounders**

|  | **Unadjusted** | **Model 1** | **Model 2** | **Model 3** |
| --- | --- | --- | --- | --- |
| PLR (per 1-SD) | 1.25 (0.92-1.68) | 1.24 (0.91-1.69) | 1.23 (0.89-1.69) | 0.99 (0.71-1.38) |
| Quartile of PLR |  |  |  |  |
| Q1 | 0.47 (0.17-1.30) | 0.45 (0.16-1.28) | 0.48 (0.17-1.41) | 0.76 (0.24-2.39) |
| Q2 | 0.47 (0.17-1.30) | 0.48 (1.67-1.38) | 0.48 (0.17-1.37) | 0.65 (0.21-2.03) |
| Q3 | 1.20 (0.52-2.74) | 1.09 (0.46-2.58) | 1.05 (0.44-2.52) | 1.25 (0.46-3.38) |
| Q4 | 1 (reference) | 1 (reference) | 1 (reference) | 1 (reference) |
| NLR (per 1-SD) | 1.23 (0.91-1.65) | 1.28 (0.94-1.75) | 1.30 (0.95-1.77) | 1.11 (0.79-1.54) |
| Quartile of NLR |  |  |  |  |
| Q1 | 0.78 (0.31-1.98) | 0.64 (0.24-1.71) | 0.61 (0.23-1.64) | 0.86 (0.29-2.52) |
| Q2 | 0.80 (0.32-2.03) | 0.71 (0.27-1.88) | 0.66 (0.25-1.77) | 0.67 (0.22-2.02) |
| Q3 | 0.78 (0.31-1.98) | 0.72 (0.27-1.88) | 0.75 (0.28-1.96) | 0.74 (0.25-2.22) |
| Q4 | 1 (reference) | 1 (reference) | 1 (reference) | 1 (reference) |
| LMR (per 1-SD) | 1.02 (0.73-1.42) | 1.07 (0.75-1.53) | 1.05 (0.73-1.51) | 1.26 (0.84-1.87) |
| Quartile of LMR |  |  |  |  |
| Q1 | 0.78 (0.33-1.81) | 0.71 (0.28-1.75) | 0.71 (0.28-1.78) | 0.41 (0.14-1.20) |
| Q2 | 0.53 (0.21-1.34) | 0.53 (0.20-1.39) | 0.52 (0.20-1.39) | 0.35 (0.12-1.10) |
| Q3 | 0.30 (0.10-0.87) | 0.28 (0.09-0.82) | 0.28 (0.10-0.85) | 0.18 (0.05-0.59) |
| Q4 | 1 (reference) | 1 (reference) | 1 (reference) | 1 (reference) |
| CRP (per 1-SD) | 1.00 (0.72-1.40) | 0.97 (0.68-1.38) | 0.95 (0.66-1.36) | 1.17 (0.79-1.74) |
| Quartile of CRP |  |  |  |  |
| Q1 | 0.85 (0.29-2.43) | 1.01 (0.34-3.01) | 1.03 (0.34-3.12) | 0.43 (0.12-1.61) |
| Q2 | 1.25 (0.47-3.32) | 1.27 (0.47-3.46) | 1.39 (0.50-3.86) | 1.16 (0.36-3.74) |
| Q3 | 1.72 (0.68-4.37) | 1.31 (0.50-3.45) | 1.42 (0.52-3.83) | 1.82 (0.58-5.72) |
| Q4 | 1 (reference) | 1 (reference) | 1 (reference) | 1 (reference) |

**Notes:** Data are presented as odds ratios (95% confidential intervals). PLR, NLR, LMR, CRP were treated as both categorical variables (using quartile cutoff points) and continuous variables (per 1-SD), separately.

Q stands for PLR, NLR, LMR, CRP: Q1 is the lowest quartile and Q4 is the highest quartile. Cutoffs for PLR are Q1<68.2, Q2 68.2-89.3, Q3 89.3-115.3, Q4>115.3. Cutoffs for NLR are Q1<1.5, Q2 1.5-1.9, Q3 1.9-2.5, Q4>2.5. Cutoffs for LMR are Q1<3.3, Q2 3.3-4.3, Q3 4.3-5.4, Q4>5.4. Cutoffs for CRP are Q1<1.5, Q2 1.5-2.1, Q3 2.1-3.2, Q4>3.2.

Model 1: adjusted for age and gender. Model 2: adjusted for age, gender, coronary heart disease, and cognitive impairment. Model 3: adjusted for age, gender, coronary heart disease, cognitive impairment, albumin, HDL-C, and BMI.

**Abbreviations:** CRP, C-reactive protein; EWGSOP2, the updated version of European Working Group on Sarcopenia in Older People; LMR, lymphocyte-to-monocyte ratio; NLR, neutrophil-to-lymphocyte ratio; PLR, platelet-to-lymphocyte ratio; SD, standard deviation.
