# Supplementary material for: Inflammatory indexes are not associated with sarcopenia in Chinese community-dwelling older people: a cross-sectional study
Source: BMC Geriatr. 2020 Nov 7;20:457. doi: 10.1186/s12877-020-01857-5 (PMC7648963; doi:10.1186/s12877-020-01857-5)
Supplement: Supplementary file 7 — Additional file 7 Table S7. Association between PLR, NLR, LMR, CRP, and IWGS-defined sarcopenia according to Logistic Regression Models adjusted for potential confounders. [file 12877_2020_1857_MOESM7_ESM.docx]

**Supplementary Table 7. Association between PLR, NLR, LMR, CRP, and IWGS-defined sarcopenia according to Logistic Regression Models adjusted for potential confounders**

|  | **Unadjusted** | **Model 1** | **Model 2** | **Model 3** |
| --- | --- | --- | --- | --- |
| PLR (per 1-SD) | 1.15 (0.92-1.43) | 1.14 (0.90-1.44) | 1.13 (0.89-1.44) | 1.04 (0.81-1.33) |
| Quartile of PLR |  |  |  |  |
| Q1 | 0.90 (0.47-1.72) | 0.88 (0.44-1.78) | 0.89 (0.44-1.80) | 1.06 (0.51-2.18) |
| Q2 | 0.75 (0.38-1.46) | 0.76 (0.38-1.55) | 0.77 (0.38-1.56) | 0.87 (0.42-1.79) |
| Q3 | 1.17 (0.62-2.20) | 1.06 (0.54-2.11) | 1.07 (0.54-2.13) | 1.14 (0.56-2.32) |
| Q4 | 1 (reference) | 1 (reference) | 1 (reference) | 1 (reference) |
| NLR (per 1-SD) | 0.96 (0.76-1.22) | 0.99 (0.77-1.27) | 0.98 (0.77-1.26) | 0.92 (0.71-1.18) |
| Quartile of NLR |  |  |  |  |
| Q1 | 1.33 (0.70-2.49) | 1.17 (0.59-2.30) | 1.17 (0.59-2.30) | 1.34 (0.67-2.69) |
| Q2 | 0.79 (0.40-1.55) | 0.70 (0.34-1.44) | 0.70 (0.34-1.44) | 0.74 (0.35-1.55) |
| Q3 | 0.87 (0.45-1.69) | 0.80 (0.39-1.62) | 0.80 (0.39-1.62) | 0.85 (0.41-1.75) |
| Q4 | 1 (reference) | 1 (reference) | 1 (reference) | 1 (reference) |
| LMR (per 1-SD) | 0.98 (0.78-1.24) | 1.02 (0.79-1.32) | 1.02 (0.79-1.32) | 1.07 (0.83-1.39) |
| Quartile of LMR |  |  |  |  |
| Q1 | 0.84 (0.44-1.58) | 0.76 (0.38-1.53) | 0.76 (0.38-1.52) | 0.64 (0.31-1.31) |
| Q2 | 0.57 (0.29-1.11) | 0.57 (0.28-1.17) | 0.56 (0.27-1.16) | 0.53 (0.25-1.11) |
| Q3 | 0.67 (0.36-1.27) | 0.62 (0.31-1.23) | 0.62 (0.31-1.23) | 0.57 (0.28-1.14) |
| Q4 | 1 (reference) | 1 (reference) | 1 (reference) | 1 (reference) |
| CRP (per 1-SD) | 0.96 (0.76-1.22) | 0.92 (0.78-1.19) | 0.92 (0.71-1.19) | 0.98 (0.75-1.28) |
| Quartile of CRP |  |  |  |  |
| Q1 | 0.97 (0.49-1.96) | 1.20 (0.57-2.52) | 1.20 (0.57-2.52) | 0.88 (0.40-1.94) |
| Q2 | 1.17 (0.59-2.30) | 1.20 (0.58-2.46) | 1.19 (0.58-2.45) | 1.12 (0.53-2.35) |
| Q3 | 2.00 (1.04-3.82) | 1.59 (0.79-3.20) | 1.57 (0.78-3.18) | 1.65 (0.79-3.42) |
| Q4 | 1 (reference) | 1 (reference) | 1 (reference) | 1 (reference) |

**Notes:** Data are presented as odds ratios (95% confidential intervals). PLR, NLR, LMR, CRP were treated as both categorical variables (using quartile cutoff points) and continuous variables (per 1-SD), separately.

Q stands for PLR, NLR, LMR, CRP: Q1 is the lowest quartile and Q4 is the highest quartile. Cutoffs for PLR are Q1<68.2, Q2 68.2-89.3, Q3 89.3-115.3, Q4>115.3. Cutoffs for NLR are Q1<1.5, Q2 1.5-1.9, Q3 1.9-2.5, Q4>2.5. Cutoffs for LMR are Q1<3.3, Q2 3.3-4.3, Q3 4.3-5.4, Q4>5.4. Cutoffs for CRP are Q1<1.5, Q2 1.5-2.1, Q3 2.1-3.2, Q4>3.2.

Model 1: adjusted for age and gender. Model 2: adjusted for age, gender, coronary heart disease, and cognitive impairment. Model 3: adjusted for age, gender, coronary heart disease, cognitive impairment, albumin, HDL-C, and BMI.

**Abbreviations:** CRP, C-reactive protein; IWGS, International Working Group on Sarcopenia; LMR, lymphocyte-to-monocyte ratio; NLR, neutrophil-to-lymphocyte ratio; PLR, platelet-to-lymphocyte ratio; SD, standard deviation.
