# Supplementary material for: Inflammatory indexes are not associated with sarcopenia in Chinese community-dwelling older people: a cross-sectional study
Source: BMC Geriatr. 2020 Nov 7;20:457. doi: 10.1186/s12877-020-01857-5 (PMC7648963; doi:10.1186/s12877-020-01857-5)
Supplement: Supplementary file 8 — Additional file 8 Table S8. Association between PLR, NLR, LMR, CRP, and FNIH-defined sarcopenia according to Logistic Regression Models adjusted for potential confounders. [file 12877_2020_1857_MOESM8_ESM.docx]

**Supplementary Table 8. Association between PLR, NLR, LMR, CRP, and FNIH-defined sarcopenia according to Logistic Regression Models adjusted for potential confounders**

|  | **Unadjusted** | **Model 1** | **Model 2** | **Model 3** |
| --- | --- | --- | --- | --- |
| PLR (per 1-SD) | 0.90 (0.67-1.21) | 0.85 (0.62-1.17) | 0.85 (0.61-1.17) | 0.88 (0.63-1.23) |
| Quartile of PLR |  |  |  |  |
| Q1 | 1.47 (0.68-3.21) | 1.60 (0.69-3.75) | 1.64 (0.69-3.90) | 1.46 (0.60-3.57) |
| Q2 | 0.83 (0.35-1.95) | 0.91 (0.36-2.26) | 0.89 (0.35-2.24) | 0.76 (0.29-2.00) |
| Q3 | 1.37 (0.63-3.01) | 1.21 (0.51-2.86) | 1.16 (0.49-2.78) | 1.11 (0.46-2.68) |
| Q4 | 1 (reference) | 1 (reference) | 1 (reference) | 1 (reference) |
| NLR (per 1-SD) | 0.81 (0.59-1.10) | 0.82 (0.59-1.15) | 0.83 (0.59-1.16) | 0.85 (0.60-1.20) |
| Quartile of NLR |  |  |  |  |
| Q1 | 2.07 (0.91-4.73) | 1.80 (0.74-4.40) | 1.76 (0.72-4.31) | 1.64 (0.65-4.11) |
| Q2 | 1.47 (0.62-3.50) | 1.34 (0.53-3.39) | 1.28 (0.50-3.28) | 1.24 (0.47-3.26) |
| Q3 | 1.68 (0.72-3.92) | 1.64 (0.66-4.05) | 1.65 (0.67-4.09) | 1.72 (0.68-4.34) |
| Q4 | 1 (reference) | 1 (reference) | 1 (reference) | 1 (reference) |
| LMR (per 1-SD) | 1.27 (0.97-1.66) | 1.42 (1.04-1.93) | 1.40 (1.03-1.91) | 1.39 (1.01-1.90) |
| Quartile of LMR |  |  |  |  |
| Q1 | 0.35 (0.15-0.85) | 0.26 (0.10-069) | 0.26 (0.10-0.70) | 0.25 (0.09-0.71) |
| Q2 | 0.49 (0.22-1.09) | 0.45 (0.19-1.10) | 0.46 (0.19-1.11) | 0.49 (0.20-1.22) |
| Q3 | 0.93 (0.46-1.86) | 0.89 (0.41-1.91) | 0.90 (0.42-1.95) | 0.92 (0.42-2.02) |
| Q4 | 1 (reference) | 1 (reference) | 1 (reference) | 1 (reference) |
| CRP (per 1-SD) | 1.09 (0.84-1.42) | 1.05 (0.78-1.41) | 1.04 (0.77-1.40) | 1.02 (0.74-1.40) |
| Quartile of CRP |  |  |  |  |
| Q1 | 0.48 (0.19-1.19) | 0.59 (0.23-1.55) | 0.60 (0.22-1.58) | 0.64 (0.23-1.77) |
| Q2 | 0.75 (0.33-1.71) | 0.74 (0.31-1.76) | 0.78 (0.32-1.89) | 0.73 (0.29-1.81) |
| Q3 | 1.80 (0.88-3.70) | 1.33 (0.60-2.91) | 1.44 (0.64-3.22) | 1.27 (0.55-2.92) |
| Q4 | 1 (reference) | 1 (reference) | 1 (reference) | 1 (reference) |

**Notes:** Data are presented as odds ratios (95% confidential intervals). PLR, NLR, LMR, CRP were treated as both categorical variables (using quartile cutoff points) and continuous variables (per 1-SD), separately.

Q stands for PLR, NLR, LMR, CRP: Q1 is the lowest quartile and Q4 is the highest quartile. Cutoffs for PLR are Q1<68.2, Q2 68.2-89.3, Q3 89.3-115.3, Q4>115.3. Cutoffs for NLR are Q1<1.5, Q2 1.5-1.9, Q3 1.9-2.5, Q4>2.5. Cutoffs for LMR are Q1<3.3, Q2 3.3-4.3, Q3 4.3-5.4, Q4>5.4. Cutoffs for CRP are Q1<1.5, Q2 1.5-2.1, Q3 2.1-3.2, Q4>3.2.

Model 1: adjusted for age and gender. Model 2: adjusted for age, gender, coronary heart disease, and cognitive impairment. Model 3: adjusted for age, gender, coronary heart disease, cognitive impairment, albumin, HDL-C, and BMI.

**Abbreviations:** CRP, C-reactive protein; FNIH, the Foundation for the National Institutes of Health; LMR, lymphocyte-to-monocyte ratio; NLR, neutrophil-to-lymphocyte ratio; PLR, platelet-to-lymphocyte ratio; SD, standard deviation.
